# Veratrum in Cholera Morbus

**Published:** 1888-11

**Authors:** H. P. Holmes

**Affiliations:** Sycamore, Ill.


### VERATRUM IN CHOLERA MORBUS.

H. P. Holmes, M. D., Sycamore, Ill.

I was called out on the night of August 24th to see a son of the Emerald Isle, and told to hurry as his family feared he would die before I could get there.

Found him in bed, suffering with cholera morbus, bathed in a cold perspiration, very weak and badly frightened. He had been vomiting and purging for about two hours, and had severe cramping pains through the bowels and in the lower extremeties. Stools thin, green, and watery. Constant thirst and drinking large quantities of cold water. The patient urged me to do something at once or he would be dead entirely.

I had not brought my library with me,* as I had been called in a great hurry, and besides I had loaned my wheelbarrow to a neighbor. I did not have time to go to my office and study the case. What was much more fortunate for the patient, I had committed to memory in my spare moments quite a slice of the materia medica, and hastily reviewing the case I found there were so many symptoms indicative of Veratrum album, that I ventured to try it, although I fully realized it was in opposition to the well-founded belief of many of the leading members of our I. H. A.

My rule of practice is, when you find a remedy well indicated, give it high to begin with. Accordingly I made six powders of the 200th of Veratrum album and gave directions to give them every half hour until the patient was relieved, stopping them as soon as he felt like going to sleep.

I considered it a desperate case, as several such had died under the old school régime in our town, and it was a good chance to test not only the value of high potencies, but Homœopathy as well. I called to see my patient early the next morning and found him sitting up. He was free from pain, had vomited but once after I left and had had but one movement of the bowels. He said he had taken but four of the powders and as they had relieved his pain so quickly he thought they must be Morphine, and so would not take any more. I put him on placebo, to be taken every two hours, and was both surprised and delighted to see him down town marketing in the afternoon.

I think this case speaks for itself. The right to have the ability to prescribe for acute cases at the bedside is questioned by but few physicians, and they, I fear, are very apt in their discussions to confound the treatment of such cases with those of a chronic nature. In cases calling for immediate attention it seems to me a risky piece of work to either take out a library at the bedside or to go back to one's office to study it up. While the value of a "book prescription" made at such a time might be far greater than one made off-hand the chances of getting just enough embarrassed to be unable to find anything at all in a repertory would be very great. I have not, as a rule, been able to find just what I wanted when I was in a hurry; and while haste is perhaps not the best thing in a critical case, it is

*See pages 586 *et seq.*, about the use of books at the bedside.

in just those cases that one needs to be able to work rapidly and surely. Let those use their books who want or need them.

Another lesson to be learned and one that applies more to the younger members of our great school, and that is the value of strictly homœopathic treatment in the most desperate cases. "What do you do when you find a patient who is actually sick?" is very often asked of me by non-believers. It is no more necessary to resort to the strong remedies and Morphine in acute cases than in chronic ones. I have always contended that the well-indicated remedy will relieve pain in a given case better and quicker than Morphine, with this advantage, the homœopathic action is *curative* while the Morphine, as Walt Whitman says, "costs more than it comes to."

